# Immunological and non-immunological mechanisms of allergic diseases in the elderly: biological and clinical characteristics

**DOI:** 10.1186/s12979-017-0105-4

**Published:** 2017-12-20

**Authors:** Gabriele Di Lorenzo, Danilo Di Bona, Federica Belluzzo, Luigi Macchia

**Affiliations:** 1 Dipartimento BioMedico di Medicina Interna e Specialistica (Di.Bi.M.I.S.), Università di Palermo, Palermo, Italy; 2 Department of Allergy, Clinical Immunology, Emergency Medicine, and Transplants, University of Bari, Bari, Italy; 3 Dipartimento BioMedico di Medicina Interna e Specialistica (Di.Bi.M.I.S), Via del Vespro, 141, 90127 Palermo, Italy

**Keywords:** Elderly, Immunosenescence, Allergic conjunctivitis, Allergic rhinitis, Asthma, Skin diseases

## Abstract

A better hygiene, a Westernized diet, air pollution, climate changes, and other factors that influence host microbiota, a key player in the induction and maintenance of immunoregulatory circuits and tolerance, are thought to be responsible for the increase of allergic diseases observed in the last years. The increase of allergic diseases in elderly is related to the presence of other factors as several comorbidities that should interfere with the development and the type of allergic reactions. A central role is played by immunosenescence responsible for modifying response to microbiota and triggering inflamm-ageing. In addition, in elderly there is a shift from Th1 responses vs. Th2, hence favouring allergic responses. Better understanding of the mechanisms of immunosenescence and its effects on allergic inflammation will most certainly lead to improved therapy.

## Background

Immediate hypersensitivity (Type I) is the most common immunological disease. About 25% of the population in industrialized countries is affected by Type I reactions, with manifestations ranging from impairment of quality of life to severely life-threatening. They may include eczema, conjunctivitis, rhinitis, asthma and anaphylaxis. Among the causes of the rapid increase in allergies are climate, pollution, diet, and the resulting microbial colonization patterns. These factors trigger and maintain a low chronic inflammatory state that characterizes allergic diseases. Most of the studies of allergic diseases and their clinical manifestations have been conducted on children or adolescents rather than on adults aged >65 years who will represent about 25% of the population in industrialized countries within the next few years. The prevalence of allergic diseases in the elderly ranges from 5 to 10% and appears to be rising [1].

## Immunosenescence

Immunosenescence is the reduction of the immune system capabilities to face stressing agents and to maintain the homeostasis. This process contributes to the reduced resistance to infectious diseases, increased propensity to develop cancer, and more frequent autoimmune diseases observed in aged individuals. A central role in allergies is played by a compromise of the integrity of epithelial barriers, a sub-clinical chronic inflammatory condition, and an enhanced Th2 (allergic) immune response [2].

Many aspects of immune function decline with ageing, while others become more active. The main hallmarks of immunosenescence are lymphocyte sub-population imbalances (decreased naive and increased memory lymphocytes with accumulation of dysfunctional senescent cells with shortened telomeres), thymus involution with decreased new T-cell generation, hematopoietic stem cell dysfunctions [3], defects in apoptotic cell death, mitochondrial function and stress responses, and malfunctioning of immune regulatory cells. As a consequence, a senescent immune system is characterized by impaired interactions between innate and adaptive immune responses, continuous reshaping and shrinkage of the immune repertoire by persistent antigenic challenges, and chronic low-grade inflammation [4].

The most extensively studied component of the immune system with regard to immunosenescence is the T-cell population. The involution of the thymus gland begins shortly after birth, undergoes replacement by fatty tissue, and is nearly complete by 60 years of age. As a consequence, there is a reduction of circulating naive T-cells and an imbalance towards memory T-cells (CD45RO+). Additionally, the T-cell receptor repertoire diversity appears to diminish, and T helper cell activity declines [5]. Other observations of the T-cell population with ageing include reduced proliferation responses [6], a decrease in CD8+ T cell levels, a shift of Th1 to Th2 cytokine profiles upon stimulation with phorbol myristic acid, a decline in FAS-mediated T-cell apoptosis [7], and increased DR expression on T-cells. In addition, an increased proportion of FOXP3+ CD4+ T regulatory cells with intact suppression capabilities has been found in peripheral blood from elderly subjects, which may help explain the decreased T-cell activities described above. Whether any of these age-related changes is more or less pronounced in specific inflammatory disorders, such as allergic diseases or asthma, is not known.

The role of cytokines in the elderly has been debated because ageing is a dynamic process characterized by a continuous remodelling sustained by DNA repair, apoptosis, immune response, oxidation stress and inflammation. In other words, the genetic background of any subject controls immunity and inflammation and influences the chronic antigen load and the inflammation in ageing responsible for immunosenescence and hence age-related disorders.

Immunosenescence is the name given to the global age-associated immune dysfunctions [8–10]. There are several hypotheses to explain the ageing process; the same is true for immunosenescence [11, 12]. Virtually all cells of the immune system can undergo immunosenescence, which can lead to the general erosion of immune capacities. Animal and in vitro models [13] substantiate the existence of immunosenescence in humans [14].

NK cells are cytotoxic cells that play a significant role in innate defence against virus infected cells and possibly cancer. It was speculated that the cytotoxicity of NK cells is directly correlated to a successful ageing; a weaker response in also related to augmented morbidity and mortality due to infective and cardiovascular agents and to a worse response to influenza vaccination. Other aspects of NK cell function, such as the secretion of chemokines or interferon-γ (IFN-γ) in response to IL-2 also decrease in the aged. NK cells have an important role in immune surveillance, and any alterations in their function will influence susceptibility to pathogens and the control of cancer development [15].

The number and phagocyte capacity of neutrophils is well preserved in the elderly. However, certain other functional characteristics of neutrophils from elderly individuals, such as super-oxide anion production, chemotaxis, and apoptosis in response to certain stimuli, are reduced. It has been hypothesized that a reduction of the transduction ability of some receptors could be a decrease in signal transduction of certain receptors could be involved in the defective function of neutrophils with advancing age [16]. In particular, there is triggering of activating receptors such as Toll-like receptor-4 (TLR4), granulocyte macrophage colony stimulating factor (GM-CSF). Similarly, anti-apoptotic signals delivered by GM-CSF failed to rescue neutrophils from apoptosis in the elderly [16].

The number of monocytes in peripheral blood does not change substantially with age, although there is a reduced number of macrophage precursors and bone marrow macrophages. However, ageing demonstrated to affect macrophage phagocytosis, immune cells recruitment ability, ROS production and TLR function response [9]. Finally, the reduction of Class II major histocompatibility (MHC) expression is thought to be responsible for decreased antigen presentation by macrophages with age [17]. Furthermore, the hyper-production of prostaglandin E2 by activated macrophages at least partly explains the reduced surface expression of Class II MHC [18].

DCs are the major antigen-presenting cells (APC), which are considered the starter of the adaptive immune response. It has been shown that DCs retain their antigen presentation function with healthy ageing [19], whereas DCs from frail elderly people display changes in co-stimulatory molecules. In brief, impaired activation of the immune response, poorer vaccine response, higher susceptibility to infection, higher susceptibility to cancer, and greater morbidity and mortality, are explained by alterations of the NK cells, phagocytes, and DCs. Ageing is correlated with a reduced number of DC descending from myeloid precursors, and have a more efficacy and mature phenotype such as a defecting ability to generate IL-12 with age. [20, 21]. Macropino-cytosis, endo-cytosis, response to chemokines, and cytokine secretion are also impaired, probably as a consequence of decreased activation of the phosphoinositide-3 kinase pathway [22].

Immune longitudinal studies in octo- and nona-genarians performed to establish predictive factors for longevity [23–25] in the context of function, and also measuring disability parameters, favour the hypothesis that the immune risk profile (IRP) predictive of subsequent mortality seems to depend in part on CD4 < CD8, low B cells, poor proliferation response, high CD8 + CD28 cells, low native cells, cytomegalovirus (CMV) seropositivity, and expansion of CMV-specific clones. Therefore there is an interplay between the IRP, low grade inflammation, and cognitive impairment in mortality. The IRP was constituted by immune subsections consisting of a high count of CD8+ T cells, a reduced number of CD4+ T cells and CD19+ B cells, a reverted CD4-CD8 ratio, and a diminished response to concanavalin A [23]. Extensive analysis to search for associations between this IRP and various psychosocial parameters revealed that the IRP was associated only with evidence of persistent CMV infection, becoming prevalent in the very old. The accumulation of large numbers of CMV-specific CD8+ T cells [24] is found, as well as a majority of clonal expansions. In the very old an association with CMV has provided additional support for the hypothesis that CMV contributes markedly to the development of an IRP and thus constitutes a good biomarker of immunosenescence in the elderly [10]. The increase in circulating inflammatory mediators such as cytokines and acute phase proteins seems to contribute to the low-grade inflammation observed with ageing. Age-related alterations in responses to stimulation also contribute to low-grade inflammation by changing the level of pro-inflammatory mediators such as TNF-α and IL-6. Because of their association with pathological cases and chronic diseases, inflammatory mediators can also act as biomarkers or risk factors for age-associated diseases and predictors of mortality.

Both the IRP and inflammageing demonstrated being independent predictors of successful ageing and survival, suggesting that the physiological immunosenescence of T cells and low-grade inflammation are vital in late-life survival [23]. Major functions known to decrease with age are IL-2 production and T-cell proliferation [5]. This in vitro evidence would suggest an in vivo clonal expansion deficiency following antigen recognition partly explaining age-associated increased susceptibility to infections, auto-immune diseases, and cancers.

The dysfunction age-related of the immune system described above might also influence the efficacy of the vaccination in the old patient [26].

Although efficient in the great percentage of the individuals, only a small percentage of frail elderly is protected after influenza vaccination [27, 28]. This is partly due to the fact that antibodies produced by aged B cells are commonly of low affinity, providing less efficient protection compared to young individuals [29]. B-cell lymphopoiesis is also reduced, which leads to an increase in the percentage of antigen-experienced cells compared to newly produced naive B cells, parallel to the situation with T cells [30].

Recently Minciullo et al. have described the role of IL-1, IL-2, IL-6, IL-12, IL-15, IL-18, IL-22, IL-23, TNF-α, IFN-γ as pro-inflammatory cytokines, and IL-1Ra, IL-4, IL-10, TGF-β1 as anti-inflammatory cytokines, and lipoxin A4 and heat shock proteins as mediators of cytokines. They hypothesize that if inflamm-ageing is a key to understand ageing, anti-inflamm-ageing may be one of the secrets of longevity [31].

### Allergic diseases in the elderly

The allergic diseases in the elderly are driven by cell ageing at large, and by immunosenescence and tissue structure modifications typical of advanced age.

### Allergic conjunctivitis

Ocular allergy is a disease affecting the entire ocular surface including conjunctiva, lids, cornea, lachrymal gland and tear film. The spectrum of atopic eye diseases encompasses seasonal allergic conjunctivitis (SAC), perennial allergic conjunctivitis (PAC), vernal kerato-conjunctivitis (VKC), atopic kerato-conjunctivitis (AKC), atopic blepharo-conjunctivitis (ABC), and giant papillary conjunctivitis (GPC). A papillary conjunctivitis is common to these diseases, and with the exception of GPC, there is also evidence of a Type I, IgE-mediated, hypersensitivity response [32].

Very few data are available in literature regarding the prevalence, role, and management of allergic conjunctivitis in the aged population [33]. Allergic conjunctivitis affects mainly children and young adults, but an increasing number of cases is being diagnosed in the elderly. Conjunctivitis can be classified as “mild”, “moderate,” or “severe,” depending on the character of disease presentation, or according to onset and duration, it can be classified as “acute,” or “chronic,” and “recurrent”, or as “follicular” and “papillary conjunctivitis,” “cicatrising” and “non-cicatrising,” emphasizing the predominant clinical presentations. AKC is a chronic conjunctivitis, with progressive corneal vascularization and scarring [34]. The new classification for allergic conjunctivitis divides the conditions into IgE-mediated and non-IgE-mediated conjunctivitis. IgE-mediated conjunctivitis can be further divided into intermittent and persistent conjunctivitis. Persistent allergic conjunctivitis is classified into VKC and AKC. [35] The International Ocular Inflammation Society (IOIS) proposed a more comprehensive classification for conjunctivitis and blepharitis, including ocular allergy in the “non infectious, immunomediated” conjunctivitis and including both the “IgE-mediated” SAC and PAC, and “non-IgE-mediated” VKC and AKC. [36].

In Table 1 we report a schematic summary of the mechanisms and cells involved in ocular allergic diseases. The symptoms of allergic conjunctivitis were reported by 68.6% of subjects with current rhinitis, accounting for a prevalence of rhino-conjunctivitis of 20.5% (95% CI: 19.2%–21.8%) in the studied population [37].

**Table 1:** Immunoglobulin and cells involved in ocular allergic diseases

	IgE	MC	Eos	TH1	TH2	Corneal involvement
SAC	+++	+++	+	–	+	–
PAC	+++	++	+	–	+	–
VKC	+	++	+++	+	+++	+++
AKC	+	++	+++	++	+++	+++
GPC	–	+	–	+	+	+
CDC	–	–	+	+++	–	–

#### Allergic rhinitis

Allergic rhinitis (AR) is prevalent among older people, affecting around 5.4 to 10.7% of patients over 65 years old [38]. Typical symptoms of allergic rhinitis such as nasal obstruction, postnasal drip or cough may be worsened by the anatomic and physiological changes of the nose that occur with age. The ageing nose undergoes changes in all of its structural components. The fibroelastic attachments between the upper and lower cartilages of the nose fragment undergo ossification with ageing. Because of maxillary alveolar hypoplasia, the columella shortens, resulting in a droopy tip appearance [39].

Information about the effect of ageing on the changes of the nasal ciliated epithelium is very limited. The number of goblet cells decreases, resilient structures atrophy, and the basement membrane gets thicker with ageing. Human respiratory and olfactory mucosa present an age-related decrease in the intensity and extent of immunoreactivity within the nasal cells [40]. However, there is no significant age-related change in gross and electron microscopic examination of the histopathology of the mucosa of either the septum or the turbinates [39].

Few studies have addressed the impact of age on nasal airflow. The ageing mucosa is less soft and less elastic (possibly hormonal effects), which may lead to increased resistance. The results of studies on the effect of ageing on nasal mucociliary clearance (NMCC) and nasal ciliary beat frequency (NCBF) are controversial. However, a decrease in NCBF and an increase in NMCC time could have a negative effect on the efficiency of NMCC [41].

It is well known that the sense of smell diminishes with age. The mean prevalence of disturbance of olfaction on a population of U.S. residents between 53 and 97 years of age is 24.5%. The prevalence increased with age, and 62.5% of 80- to 97-year-old subjects has olfactory impairment [42]. The sense of smell comprises multiple sensations that are predominantly mediated by two independent neural systems—the olfactory and the somatosensory (trigeminal) [43].

These alterations of the nasal anatomy and physiology due directly to the normal ageing process result in symptoms of postnasal dripping, nasal drainage, sneezing, olfactory loss, and gustatory rhinitis. The other common nasal symptoms include nasal obstruction, headache, sinus pain, itching, and epistaxis. Below we report important aetiologies of nasal problems in elderly patients.

Vasomotor rhinitis, atrophic rhinitis, and gustatory rhinitis are common types of nonallergic rhinitis types that occur in older patients [44]. Gastroesophageal reflux has often been associated with vasomotor rhinitis [45]. Primary atrophic rhinitis was commonly associated with infection of *Klebsiella ozaenae.* Currently, it is more commonly seen as a result of aggressive surgery, trauma, granulomatous diseases, and radiation therapy [44].

Gustatory rhinitis is a profuse watery rhinorrhoea that may be exacerbated by eating. It is believed to arise from α-adrenergic activity stimulated by the regular use of anti-hypertensives. Allergic rhinitis and its severity decrease with age, and there are significantly fewer cases of atopy among elderly subjects (60 years or older) compared to younger individuals [46]. However, the repeatedly claimed global decline in the prevalence of allergic disorders in the elderly might be ascribed to the expected decrease in serum IgE antibodies due to an unbalance of cytokines and soluble factors involved in its production. In the assessment of serum IgE, sCD23 and Th2 type cytokine production, however, IgE serum levels did not differ in a relevant way among all the ages in non allergic subjects [47]. This was confirmed in another, similar study [48] that suggests the Type 2 cytokine pattern is not necessarily defective in old age. Data also confirmed that IL-13, a key cytokine in IgE regulation, is not impaired in old subjects. Although IL-4 has been considered the most critical cytokine linked to allergic responses and immunity against parasites, recent observations indicate that IL-13 has equal or even greater importance in those processes. IL-4 and IL-13 share several functional properties, but IL-13 can independently induce class switching and IgE secretion from human B cells. In addition, IL-13 enhances expression of CD23 and the MHC Class II antigens, and may act as a monocyte chemo-attractant [49]. Rhinitis associated symptoms seems to be milder and allergy-related parameters usually gradually decline in the long run; often, these nasal symptoms appear to be linked to the nasal eosinophils and are independent on SPT and specific IgE [50].

The increase of AR in elderly can be explained on the basis of the general hypothesis that imbalance of gut microbiota, linked to immunosnescence, influences the development of allergic diseases [51].

#### Asthma

Asthma represents a significant cause of morbidity and mortality in the elderly, while in the past it was regarded as an illness of childhood and youth. Asthma remains under-diagnosed in old people, and the percentage increases when respiratory symptoms are present. There are two kinds of old patients affected by asthma: one who had the onset of the disease in the childhood and one having encountered the symptoms in the sixth decade of life [52]. Current knowledge suggests a phenotype difference of asthma in old and young subjects, and this could potentially impact on diagnosis, assessment, and management of the disease. The same diagnostic tests and clinical findings applied in youth are used to diagnose asthma in the elderly, but interpreting clinical data becomes more difficult [53]. Asthma in the elderly is broadly divided into patients with long-standing disease present from childhood, and late-onset disease describing those developing symptoms following the sixth decade of life. The diagnosis in the second case could be difficult due to the presence of similar diseases with almost few equal symptoms, which have more prevalence in the elderly such as chronic obstructive lung disease (COPD) or heart failure [54].

Although the shortness of breath, chest tightness, cough, and wheezing that characterize asthma in young people are present in the elderly, mimicry by congestive heart failure, chronic obstructive pulmonary disease, ischemic heart disease, gastroeosphageal reflux, pulmonary emboli, recurrent aspiration, respiratory track cancer, and laryngeal dysfunction make the diagnosis a challenge. Angiotensin converting enzyme inhibitor-induced cough is also a frequent masquerade. Older individuals often have poorer perception of airway obstructive symptoms and are less likely to report them. They may falsely attribute symptoms to “getting older” and avoid activities, including exercise, that trigger asthma symptoms. For many years asthma in older patients was characterized as non-atopic [55]. However, in the past two decades, data from large populations or from studies incorporating data from multiple sites of asthma care, demonstrate that some older patients with asthma are also atopic (demonstrated either by serum evaluation or skin prick testing). Busse et al., demonstrated a higher allergic sensitization rate among asthmatics, at 62.5%, compared to 38.8% in the general population in subjects ≥55 years of age [56].

Sensitization to indoor allergens alone, rather than indoor and outdoor allergens, has been suggested as potentially more important to asthma in older patients [1]. Distinguishing between asthma and COPD frequently becomes an issue when airway obstruction is evaluated in the elderly. In a study focusing on the lung function and inflammatory differences between asthma and COPD, it was observed that in the asthma subjects there was significantly more allergic sensitivity, higher values for alveolus-capillary diffusion of carbon monoxide, greater increases in forced expiratory volume in the 1st second, following bronchodilator or corticosteroids, and more eosinophils in peripheral blood, bronchoalveolar lavage and sputum [53]. However, it is also likely that an overlap syndrome exists for some patients in which features of asthma and COPD are both present, but this subset of patients has yet to be carefully examined and is typically excluded from investigation studies. The “Dutch Hypothesis” is an interesting view of asthma and COPD that proposes there is one common obstructive lung disease that includes both asthma and COPD [57]. This hypothesis suggests that a common genetic predisposition for obstructive lung disease exists, and that asthma and COPD differ with respect to the lung exposures (allergen versus tobacco smoke) that trigger and drive the disorder towards airway obstruction. However, this hypothesis remains controversial because it cannot fully explain some of the differences observed between asthma and COPD [58].

Because viral infections of the upper respiratory tract trigger the majority of asthma exacerbations, age-related decline in antiviral responses affects the associated morbidity and mortality [2].

#### Skin diseases

Ageing contributes progressively to a loss of structural integrity and physiological function of the skin. Although skin is incredibly durable, it is affected by ageing, like all other organ systems [59]. The synergistic effects of biological, environmental, mechanical ageing, and miscellaneous factors including diet, sleep patterns, morbidity, and mental health over the human lifespan combine to cause deterioration of the cutaneous barrier and the structural integrity of the skin. Hormonal changes that also play a role in the ageing of skin, especially in females, lead to earlier signs of ageing for women [60]. However, skin ageing can also produce significant morbidity, pervasive dryness and itching, and increased risk of numerous skin diseases, including cutaneous malignancy. Most people over 65 have at least one skin disorder, and many have two or more. Cell numbers in the epidermis are reduced in older adults [61]. Keratinocytes change shape, becoming shorter and fatter, as skin ages [62], whereas corneocytes in aged skin become bigger as a result of decreased epidermal turnover [61]. Epidermal turnover time increases in aged skin [63].

Since permeability barrier function in ageing epidermis does not appear to be impaired under basal conditions, it has been generally assumed that barrier function does not alter significantly with ageing [64]. Recovery of barrier function in aged subjects was also dramatically different. Only 15% of those older than 80 years had recovered barrier function at 24 h, compared to 50% of the younger group [65]. The findings reveal a profound change in barrier integrity even though barrier function under normal conditions appears normal. A lack of functional reserve is exposed when the epidermal permeability barrier is under stress [65]. Although the lipid composition of aged skin is not significantly altered, the global lipid content of aged skin is reduced [65]. Total lipid content in aged skin decreased as much as 65% [66].

The flattened dermal–epidermal junction, with its reduced interdigitation between layers, results in less resistance to shearing forces and an increased vulnerability to insult [67]. Dermal thickness decreases with age [68], with a decrease in vascularity and cellularity. There is also a decrease in the number of mast cells and fibroblasts [69]. However, ageing is inevitably associated with a decrease in collagen turnover (due to a decrease in fibroblasts and their collagen synthesis) as well as elastin [69]. The loss of molecular integrity of the dermis leads to increased rigidity, decreased torsion extensibility and diminished elasticity (eroding faster in females than in males), with a concomitant increase in vulnerability to tear type injuries [67].

The overall volume of subcutaneous fat typically diminishes with age, although the proportion of body fat increases until approximately age 70 [67]. Contact dermatitis is common in the elderly population (particularly allergy-type reactions) [2]. Reduced ability to mount a delayed-type hypersensitivity reaction in the elderly decreases individual susceptibility to allergic contact sensitivity due to a reduction in numbers of Langerhans cells [70], decreased T-cells, and diminished vascular reactivity [71]. However, decades of potential sensitization [72] and an increased level of exposure maintain a presence of allergic contact sensitivity in the geriatric population [73]. The most common culprit in allergic contact sensitivity is topical medications [74]. As many as 81% of patients being treated for chronic leg ulcers exhibit allergic reactions to topical medications. Patch testing before the use of topical medications may be beneficial, especially within high-risk populations like those being treated for dermatitis or ulceration of the lower extremities [75]. Testing should include medication and dressings, as well as dental prostheses, and medications for ocular disease [76]. In the elderly a generalized allergic rash is far more likely to be due to drugs rather than to be food-related. Occasionally an agent increases the patient’s sensitivity to the sun in a phototoxic (photoirritant) reaction, or produces a hypersensitivity reaction upon sun exposure [77].

However, it may be relevant that with ageing, in the skin, total IgE production increases with the decrease in IgE levels towards specific allergens [78].

## Conclusion

In the last years the prevalence of allergic diseases in general population is increased because of environmental changes such as the a better hygiene, a Westernized diet, air pollution, climate changes, and other factors that influence host microbiota. Microbiota is a key player in the induction and maintenance of immunoregulatory circuits and tolerance and its changes can determine immune dysregulation, and subsequent low-grade chronic inflammation, which is a common pathogenic mechanism in several diseases including the allergic ones. Additional factors are responsible for the increase of allergic diseases in elderly as the presence of several comorbidities that should interfere with the development and the type of allergic reactions. However, immunosenescence plays a central role by modifying response to microbiota and triggering inflamm-ageing. In addition, in elderly there is a shift from Th1 responses vs. Th2, hence favouring allergic responses. Better understanding of the mechanisms of immunosenescence and its effects on allergic inflammation will most certainly lead to improved therapy [79–81]. Optimal treatment of elderly patients requires an alliance between the patient, geriatrician, and allergist.

## Abbreviations

ABC: Atopic blepharoconjunctivitis
AKC: Atopic keratoconjunctivitis
APC: Antigen-presenting cells
AR: Allergic rhinitis
CMV: Cytomegalovirus
COPD: Chronic obstructive pulmonary disease
DC: Dendritic Cell
GM-CSF: Granulocyte-macrophage colony stimulating factor
GPC: Giant papillary conjunctivitis
IFN-γ: Interferon-γ
IOIS: International Ocular Inflammation Society
IRP: Immune risk profile
MCH: Major histocompatibility complex
NCBF: Nasal ciliary beat frequency
NK: Natural Killer
NMCC: Nasal mucociliary clearance
PAC: Perennial allergic conjunctivitis
SAC: Seasonal allergic conjunctivitis
Th: T helper
TLR4: Toll-like receptor-4
VKC: Vernal keratoconjunctivitis
